# Response to Dr Fried & Dr Kievit, and Dr Malhi *et al.*

**DOI:** 10.1038/mp.2016.9

**Published:** 2016-02-23

**Authors:** L Schmaal, D J Veltman, T G M van Erp, P G Sämann, T Frodl, N Jahanshad, E Loehrer, M W Vernooij, W J Niessen, M A Ikram, K Wittfeld, H J Grabe, A Block, K Hegenscheid, D Hoehn, M Czisch, J Lagopoulos, S N Hatton, I B Hickie, R Goya-Maldonado, B Krämer, O Gruber, B Couvy-Duchesne, M E Rentería, L T Strike, M J Wright, G I de Zubicaray, K L McMahon, S E Medland, N A Gillespie, G B Hall, L S van Velzen, M-J van Tol, N J van der Wee, I M Veer, H Walter, E Schramm, C Normann, D Schoepf, C Konrad, B Zurowski, A M McIntosh, H C Whalley, J E Sussmann, B R Godlewska, F H Fischer, B W J H Penninx, P M Thompson, D P Hibar

**Affiliations:** 1 Department of Psychiatry and Neuroscience Campus Amsterdam, VU University Medical Center, Amsterdam, The Netherlands; 2 Department of Psychiatry and Human Behavior, University of California, Irvine, CA, USA; 3 Max Planck Institute of Psychiatry, Neuroimaging Research Group, Munich, Germany; 4 Department of Psychiatry and Psychotherapy, Otto von Guericke University of Magdeburg, Magdeburg, Germany; 5 Department of Psychiatry, Trinity College, University of Dublin, Dublin, Ireland; 6 Imaging Genetics Center, Department of Neurology, Keck School of Medicine, University of Southern California, Marina del Rey, CA, USA; 7 Department of Environmental Health, Harvard T.H. Chan School of Public Health, Boston, MS, USA; 8 Department of Epidemiology, Erasmus MC University Medical Center, Rotterdam, The Netherlands; 9 Department of Radiology, Erasmus MC University Medical Center, Rotterdam, The Netherlands; 10 Department of Medical Informatics, Erasmus MC University Medical Center, Rotterdam, The Netherlands; 11 Faculty of Applied Sciences, Delft University of Technology, Delft, The Netherlands; 12 Department of Neurology, Erasmus MC University Medical Center, Rotterdam, The Netherlands; 13 German Center for Neurodegenerative Diseases (DZNE), Rostock/Greifswald, Germany; 14 Department of Psychiatry and Psychotherapy, University Medicine Greifswald, Greifswald, Germany; 15 Helios Hospital Stralsund, Stralsund, Germany; 16 Institute of Diagnostic Radiology and Neuroradiology, University Medicine Greifswald, Greifswald, Germany; 17 Clinical Research Unit, Brain and Mind Centre, University of Sydney, Camperdown, NSW, Australia; 18 Center for Translational Research in Systems Neuroscience and Psychiatry, Department of Psychiatry and Psychotherapy, University Medical Center (UMG), Gerog-August-University, Goettingen, Germany; 19 Section for Experimental Psychopathology and Neuroimaging, Department of General Psychiatry, Heidelberg University Hospital, Heidelberg, Germany; 20 Queensland Brain Institute, The University of Queensland, Brisbane, QLD, Australia; 21 Center for Advanced Imaging, The University of Queensland, Brisbane, QLD, Australia; 22 Queensland Institute of Medical Research Berghofer, Brisbane, QLD, Australia; 23 Department of Genetic Epidemiology, Queensland Institute of Medical Research Berghofer, Brisbane, QLD, Australia; 24 Faculty of Health, The Institute of Health and Biomedical Innovation, Queensland University of Technology (QUT), Brisbane, QLD, Australia; 25 Department of Quantitative Genetics, Queensland Institute of Medical Research Berghofer, Brisbane, QLD, Australia; 26 Virginia Institute for Psychiatric and Behavioral Genetics, Virginia Commonwealth University, Richmond, VA, USA; 27 Department of Psychology, Neuroscience and Behaviour, McMaster University, Hamilton, ON, Canada; 28 Imaging Research Centre, St. Joseph's Healthcare Hamilton, Hamilton, ON, Canada; 29 University of Groningen, University Medical Center Groningen, Department of Neuroscience, Neuroimaging Center, Groningen, The Netherlands; 30 Department of Psychiatry, Leiden University Medical Center, Leiden University, Leiden, The Netherlands; 31 Leiden Institute for Brain and Cognition, Leiden, The Netherlands; 32 Division of Mind and Brain Research, Department of Psychiatry and Psychotherapy, Charité Universitätsmedizin Berlin, Berlin, Germany; 33 Department of Psychiatry and Psychotherapy, University Medical Center Freiburg, Freiburg, Germany; 34 Psychiatric University Clinic, Basel, Switzerland; 35 Department of Psychiatry, University of Bonn, Bonn, Germany; 36 Department of Psychiatry and Psychotherapy, Agaplesion Diakoniklinikum, Rotenburg, Germany; 37 Department of Psychiatry and Psychotherapy, Philipps-University Marburg, Marburg, Germany; 38 Center for Integrative Psychiatry, University of Lübeck, Lübeck, Germany; 39 Division of Psychiatry, University of Edinburgh, UK; 40 Centre for Cognitive Ageing and Cognitive Epidemiology, University of Edinburgh, UK; 41 Department of Psychiatry, Warneford Hospital, Oxford, UK; 42 Department of Psychosomatic Medicine, Center for Internal Medicine and Dermatology, Charité Universitätsmedizin, Berlin, Germany; 43 Institute for Social Medicine, Epidemology and Health Economics, Charité Universitätsmedizin, Berlin, Germany

We thank Dr. Fried & Dr. Kievit, and Dr. Malhi and colleagues for their insightful comments. Here we further clarify the design and outcome of our meta-analysis of subcortical volume differences between patients with major depressive disorder (MDD) and controls.

Fried and Kievit^1^ and Malhi *et al.*^2^ commend the collaborative achievement of the Enhancing NeuroImaging Genetics through Meta-Analysis (ENIGMA) MDD consortium in analyzing sample sizes unprecedented in neuroimaging of depression. They also raise some concerns and provide additional interpretations of our results. Malhi *et al.* suggest that we should better exploit this vast data set to study the heterogeneity of MDD. MDD is a heterogeneous disorder, and the scope and extent of brain alterations depends on specific clinical characteristics of the sample studied. That is why we reported meta-analytic results of subcortical volume differences in depressed patients stratified by stage of illness (first episode versus recurrent) and age of onset (early versus late).^3^ Compared to controls, hippocampal volume was lower in recurrent and early-onset patients, on an average, but not in first-episode patients or patients diagnosed after age 21. These findings indeed raise interesting questions: for instance, whether hippocampal volume reductions are detectable in early-onset *first*-episode patients. Malhi *et al.* suggest partitioning our sample into finer subgroups to study these interactions. We agree that it is important to analyze between-subject differences across these complex interactions, but our study design did not allow for delineating these more complex interactions (for example, diagnosis x recurrence x age of onset). Our meta-analysis comprised standardized processing, statistics and quality control to harmonize methods in a sample of unprecedented size. In a mega-analysis, one can access all participant-level data. By contrast, our initial meta-analysis distributed work efficiently across many sites, and we meta-analyzed summary statistics at a central site. Coordinated analyses at this scale must consider what is feasible and achievable, to motivate more fine-grained analysis. So far, partitioning patients into fine-grained subgroups has not been feasible in most individual samples, as the numbers per cell quickly become too small to assess more complex clinical interactions. As ENIGMA grows, several analyses are showing consistency worldwide. A mega-analysis may be feasible with a subsample of participating sites who meet legal and ethical requirements for the sharing of individual subject data to a central site.

Our study is the largest meta-analysis to date and reveals the profile of subcortical volume alterations in MDD, and some factors that affect it. Brain structure was consistently altered across: (1) MDD patients residing in the community or primary care, and (2) patients recruited from specialized mental health services, many of whom had more severe and recurrent MDD. Unlike smaller studies, we found no consistent subcortical brain alterations beyond the hippocampus, and even this was observed only in specific patient subgroups: this is, unquestionably, new information from a worldwide sample offering very high power. As Fried and Kievit note, subcortical abnormalities in MDD are moderate, but consistent. Past claims reporting smaller volumes of (for example) the amygdala in MDD patients were not robust across the cohorts we analyzed. As with any other study, ours has limitations. Not all individual studies had detailed information on duration, number of episodes and treatment history. When combining already collected data across worldwide samples, data collection protocols are not prospectively harmonized. Clinical assessments therefore differed across studies, which limits the analysis of sources of heterogeneity. For instance, different instruments were used to assess depression symptom severity across the studies included in the meta-analysis. New subprojects were recently initiated within our ENIGMA MDD consortium specifically focused on how severity impacts neural changes in MDD, which intend to (1) establish a common metric for depressive symptoms for various questionnaires, and (2) explore different ways of defining symptom and disease severity and their association with brain measures. Regarding the effects of antidepressants (cf., the letter to the editor by Malhi *et al.*), a cross-sectional study design such as ours cannot determine how antidepressant medication affects brain structure. Interventional studies comparing patients pre- and post-treatment are required to establish how antidepressants affect brain structure.

Fried and Kievit rightly note that hippocampal volume reduction in the MDD group and its subgroups is known to be small (Cohen's *d* between −0.14 and −0.21) and not specific to MDD. As our colleagues found in the ENIGMA Schizophrenia Working Group,^4^ larger effects are observed in schizophrenia, motivating cross-disorder comparisons across ENIGMA eventually. However, our finding is robust: the hippocampus was consistently smaller, on average, across a large number of samples encompassing the broad heterogeneity of MDD (*I*^2^ scores showed low heterogeneity of findings across studies). Smaller hippocampal volume has been associated with executive function impairments,^5^ learning and memory deficits^6^ and poorer treatment response^7^ in MDD; so the hippocampal volume reduction is important despite its small effect size. Establishing the degree of hippocampal volume difference in MDD, and its modulators, with this precision is crucial, as the disorder affects billions of people worldwide.

Indeed we did not estimate any form of classification accuracy. Researchers in the field of neuroimaging already realize that no single univariate data point differentiates MDD patients from controls. If classification were the goal, one could include other subcortical regions whose effects do not reach the significance threshold, but within a multivariate analysis could boost classification accuracy. Moreover, cortical regions or other imaging measures could be included. Ultimately, consortia such as ENIGMA may discover multivariate patterns predictive of diagnosis, but progress is unlikely without first publishing studies of measures that are easier to harmonize. It is widely known that findings based on group-level (univariate and mass-univariate) approaches may not offer sufficient predictive value for individual patients within a multivariate classification approach. Measures from future ENIGMA MDD projects studying cortical thickness, surface area, shape, hippocampal subfields, diffusion tensor imaging or functional measures may help multivariate prediction methods, eventually.^8^ Moreover, the harmonization of processing, statistics and quality control protocols across ENIGMA disease working groups will eventually allow classification across different psychiatric disorders.^9^

Fried and Kievit discuss some alternative mechanisms that may drive hippocampal volume reduction in MDD. In our original paper, we did not claim 'that depression causes structural changes' (cf. letter to the editor by Fried and Kievit). In fact, not only patients with recurrent episodes, but also the group with early age of onset (consisting of almost 50% of first-episode patients) showed smaller hippocampal volumes; so structural changes are not merely a consequence of depression. We speculated that hippocampal volume reductions may be promoted by a chronic hyperactivity of the hypothalamic−pituitary−adrenal axis via remodelling and downregulation of growth factors including brain-derived neurotrophic factor, associated with (chronic) stress.^10^ Stressors include multiple episodes of depression, early-life stress and a family history of depression, which are all linked to early-onset depression,^11, 12, 13^ higher risk for recurrent depression,^14, 15, 16^ an overactive hypothalamic−pituitary−adrenal axis^17, 18^ and smaller hippocampal volume.^19^ As we stated in our article, smaller hippocampal volume may even be a risk factor for depression: 'morphological hippocampal alterations may represent risk markers for depression, recurrence and chronicity' and 'Clearly, there is a continued need for longitudinal studies tracking hippocampal volume changes over the disease course, to further elucidate whether hippocampal abnormalities result from prolonged duration of chronic stress (i.e. 'scarring'), represent a vulnerability factor for MDD, or both', which agrees with Fried and Kievit.

## Consortium members

The members of the ENIGMA-Major Depressive Disorder Working Group consortium are listed at http://enigma.ini.usc.edu/ongoing/enigma-mdd-working-group/.
